# Disease burden and the role of pharmacogenomics in African populations

**DOI:** 10.1017/gheg.2016.21

**Published:** 2017-02-03

**Authors:** K. L. Mpye, A. Matimba, K. Dzobo, S. Chirikure, A. Wonkam, C. Dandara

**Affiliations:** 1 Division of Human Genetics, Department of Pathology, Faculty of Health Sciences, Institute of Infectious Diseases and Molecular Medicine, University of Cape Town, Anzio Road, Observatory, 7925, Cape Town, South Africa; 2 Department of Clinical Pharmacology, College of Health Sciences, University of Zimbabwe, P.O. Box A178, Avondale, Harare, Zimbabwe; 3 International Centre for Genetic Engineering and Biotechnology (ICGEB), Cape Town Component, Wernher and Beit Building (South), UCT Campus, Anzio Road, Observatory, 7925, Cape Town, South Africa; 4 Division of Medical Biochemistry, Faculty of Health Sciences, University of Cape Town, Observatory, 7925, Cape Town, South Africa; 5 Department of Archaeology, University of Cape Town, Cape Town, Rondebosch 7701, South Africa

**Keywords:** Disease burden, drug metabolizing enzymes, genetic variation, pharmacogenetics, pharmacogenomics, sub-Saharan Africa

## Abstract

**Background.:**

The burden of communicable and non-communicable diseases in Sub-Saharan Africa poses a challenge in achieving quality healthcare. Although therapeutic drugs have generally improved health, their efficacy differs from individual to individual. Variability in treatment response is mainly because of genetic variants that affect the pharmacokinetics and pharmacodynamics of drugs.

**Method.:**

The intersection of disease burden and therapeutic intervention is reviewed, and the status of pharmacogenomics knowledge in African populations is explored.

**Results.:**

The most commonly studied variants with pharmacogenomics relevance are discussed, especially in genes coding for enzymes that affect the response to drugs used for HIV, malaria, sickle cell disease and cardiovascular diseases.

**Conclusions.:**

The genetically diverse African population is likely to benefit from a pharmacogenomics-based healthcare approach, especially with respect to reduction of drug side effects, and separation of responders and non-responders leading to optimized drug choices and doses for each patient.

## Disease burden in Africa and challenges with therapeutic drug combinations

One of the most notable health achievements this millennium is that patients around the world are now living longer than ever before. According to the Global Burden of Disease 2013 report, in most parts of the world, ‘*Eating too much has overtaken hunger as a leading risk factor for illness*’. However, in sub-Saharan Africa, communicable, maternal, nutritional and infant diseases continue to ravage its population. Of the nearly 200 million cases of malaria reported globally in 2013, the death burden was heaviest in the African region, where an estimated 90% of all malaria cases resulted in death [1]. An estimated 36.7 million (34.0–39.8 million) people were living with HIV at the end of 2015, with sub-Saharan Africa remaining the most severely affected, accounting for at least 69% of people living with HIV worldwide. Table 1 summarizes the top 10 leading causes of death in Africa, with HIV ranked top.

**Table 1.** *Leading causes of death in Africa* (World Health Organization's Global Health Estimates, 2012)

	Disease	Number of deaths per year
1	HIV/AIDS	1 108 000
2	Lower respiratory tract infections (target lungs and airways)	1 101 000
3	Diarrheal diseases	644 000
4	Malaria	568 000
5	Stroke	427 000
6	Preterm birth complications	393 000
7	Birth asphyxiation and trauma	356 000
8	Malnutrition	307 000
9	Coronary heart disease	293 000
10	Meningitis	260 000

https://africacheck.org/factsheets/factsheet-the-leading-causes-of-death-in-africa/ (accessed 18 June 2016).

Of the total global disease burden, at least 25% occurs in sub-Saharan Africa, which has only 3% of the health workforce. This disproportionate disease burden faced by sub-Saharan Africa leads to major challenges in patient management. Additionally, patients present with co-morbidities that further complicate disease management and outcome [2]. Thus, therapeutic drug prescriptions need to take into account the interactions and counter-effects of drugs prescribed to manage co-morbidities. For example, HIV/TB co-infections are a common occurrence, presenting challenges in prescribing multiple drugs, which do not frequently arise in western countries, where these diseases rarely occur together. HIV/malaria co-treatment is also a common healthcare challenge in most African countries. Sub-Saharan Africa is the most intensely affected region, accounting for 90% of malaria deaths, more than 69% of the global HIV-positive population, 28% of all TB cases and an increasing burden of diabetes and cancer.

Managing interactions among the therapeutic drugs used to treat and manage HIV, TB, malaria, diarrheal disease and emerging non-communicable diseases require an integrated knowledge of pharmacogenomics. A major challenge is that African health systems are in transition; and in addition to concerns about conventional drugs, the co-use of herbal medicinal compounds in disease management and treatment must also be considered, which has implications for drug–drug and drug–herb interactions [3, 4].

The disease burden data for Africa are not uniform; there is a wide diversity of prevalence and mortality rates between African countries or geographical regions. For example, HIV prevalence data for 2011 varies from <1% in Algeria to >25% in Swaziland [5]. Therefore, there are different public health priorities or imperatives for various African countries. Different combinations of co-infections complicate the picture. In the southern African region, HIV/TB are the common co-infections of concern, and in West, Central and East Africa, co-infections involving HIV, TB, malaria and schistosomiasis have to be taken into account, making therapeutic drug management geographically dependent. Therefore, the adverse events [6] produced by a specific drug are different for patients in different regions because of other co-administered drugs or co-morbidities [7–11]. Pharmacogenomics could assist in improving the safety and reducing the toxicity of drugs through identification of individuals likely to benefit and mapping profiles for safe use.

Disease patterns among African patients calls for a better understanding of the pathways that are important determinants of drug response in this region. There is a need to generate pharmacogenomics data for Africans of various ethnicities in various regions in order to take into account the genetic diversity of the African population during drug treatment. The utility of pharmacogenomics in African populations can only be realized if many different ethnic groups are characterized with respect to genetic determinants or biomarker alleles that affect drug responses. This could be achieved through integrated programs such as the African pharmacogenomics consortium, which aims to harness the still unexploited genomic potential of African populations [12].

Most drugs developed to work based on successful use in patients with a European (Caucasian) genetic background may not adequately work for African patients. However, among African populations themselves, there exists a heterogeneous genetic background, and it is important that this is analyzed and documented. According to the Clinical Trials website (https://clinicaltrials.org), of the >200 000 registered ongoing and completed trials, <3% involve participants from African countries or participants with sub-Saharan African ancestry (Table 2). Thus, most drugs that will eventually be developed and approved in the near future will likely have limited information on the potential for efficacy and adverse drug reactions (ADRs) in African populations. In most cases, ADRs that are African-specific are only discovered after a drug has been released on the market.

**Table 2.** An overview of studies registered on ClinicalTrials.gov as of June 2016

Region	Number of studies	Percentage contribution
Africa	4874	2.4
*South Africa*	*(2108)*	
*Egypt*	*(1000)*	
*Kenya*	*(304)*	
*Uganda*	*(325)*	
*Tanzania*	*(215)*	
*Tunisia*	*(180)*	
*Zambia*	*(122)*	
*Malawi*	*(131)*	
Europe	57 313	28.0
Central America	2285	1.1
Middle East	8443	4.1
Canada	14 680	7.2
United States	89 561	43.7
North Asia	3754	1.8
Pacifica	5331	2.6
South America	6924	3.4
South Asia	3285	1.6
Southeast Asia	4189	2.0
Total	204 730	

https://clinicaltrials.gov/ct2/search/map (Accessed 30 June 2016).

This review explores the complexity of pharmacogenomics in African populations, what has been achieved and how pharmacogenomics interfaces with the other emerging -OMICs sciences, such as pharmacoepigenetics, to provide a holistic insight into the determinants of therapeutic drug responses. Decoding the genomes of Africans is a step toward precision medicine and understanding the genetic determinants of drug response for most world populations. Modern man originated in Africa, and therefore, most genetic traits observed in different parts of the world are traceable to African populations. The non-inclusion of African genomes in drug discovery and development research potentially leads to reduced global utility of new drugs.

## Pharmacogenomics

Pharmacogenomics is the study of how the patient genome influences responses to medicines with respect to efficacy and safety. This discipline focuses on identification of genetic variants that influence drug efficacy and safety, mostly through alterations in pharmacokinetics and pharmacodynamics. The genetic variants of interest are primarily in germline DNA (i.e. inherited); however, in cancer, somatically acquired mutations also influence the response of a patient to treatment [13–16]. The field of pharmacogenomics is taking advantage of advances in genome characterization technology and analytical power through bioinformatics, which is enabling the decoding of genetic variants affecting patient populations with specific drug-response phenotypes. Of greatest interest is the role of pharmacogenomics in drug safety, and a number of examples have demonstrated this at both individual and population levels.

Pharmacogenomics knowledge enables the realization of an emerging approach for disease treatment and prevention, precision medicine, which takes into account individual genetic variability, environmental, and lifestyle factors for each person. Prediction of efficacy and safety using genomics will form part of drug prescriptions in the future. Pre-emptive genotyping of actionable genetic variants could be a good tool to optimize pharmacotherapy in patients, especially for drugs with high pharmacogenetic variability, such as warfarin [17]. The most commonly studied genes are those coding for drug metabolizing cytochrome P450 enzymes (CYPs) and transporters, such as *ABCB1* (also referred to as *MDR-1*), *SLCO1B1* and *MRP2* [18–20]. Pharmacogenetic screening of the human leukocyte antigen (*HLA*) genes has been shown to be effective in preventing drug exposure of patients who are hypersensitive to the anti-retroviral drug, abacavir [4]. Research has also shown how chromatin structures together with variations in microRNA pathways play an important role in disease-response phenotypes [21]. Additionally, the role of environmental factors in pharmacogenetics (i.e. pharmacoepigenetics) has been demonstrated through the emergence of epigenetic drugs, affecting DNA methylation and histone modifications [22]. This underscores the need for African population-based pharmacogenomics data. Regulatory bodies such as the USA Food and Drug Administration (FDA) and the European Medicines Agency, are regularly updating guidelines for the use of genome-based therapies for the benefit of patients, making the application of genomic findings in healthcare and health systems increasingly possible. Regulatory bodies focusing on genomics are lacking among African countries, and therefore there are no clear guidelines on genomics research in Africa. It would be beneficial for African countries to fund extensive characterization and updating of genomics data, producing regular recommendations for pharmacogenomics interventions or testing. It is encouraging to note the recent formation of the ‘African pharmacogenomics consortium’ whose objectives include collaborative strategies for the discovery and curation of genetic variants that are of pharmacogenomic importance among African populations [5, 11].

## Current status and need for pharmacogenomics in Africa

Implementing pharmacogenomics in Africa has been slow because of inadequately equipped research facilities, lack of coordinated efforts, and limited expertise and leadership to apply pharmacogenomics in clinical research. Although middle-income countries such as South Africa are building resources, which support advanced genomics technologies, this is not the case for the rest of Africa, where basic facilities for genome characterization are lacking. In fact, pharmacogenomics is lagging behind in Africa compared with the USA, Europe and Asia [23], despite the obvious genetic diversity of African populations, which is complicated by a complex disease burden. However, a few studies have been conducted and their outcomes are highlighted in this review.

Clinical pharmacogenetics association studies have mostly applied candidate gene approaches using small sample sizes. However, recently, there has been a strong focus on HIV pharmacogenomics, especially evaluating efavirenz, nevirapine and stavudine with respect to the role of variation in the *CYP2B6* gene and mitochondrial DNA [24–28]. Studies on malaria have focused on polymorphisms in the *PFCRT* and *K13-propellor* parasite genes and their impact on artemisinin-based drug resistance and susceptibility [29, 30].

Pharmacogenomics is of growing importance in Africa, as more drugs are being registered and marketed for use in local populations. More than 70% of therapeutic drugs are metabolized by CYP enzymes, which exhibit genetic polymorphisms. There is a wide genetic variability in the CYP genes among African populations that translate to differences in drug response [5]. The limited data available for African-Americans have in the past been used to predict responses in African populations [11, 14]. To date, several genome-wide association studies have been applied in patient cohorts to confirm known markers and to identify novel common genetic variations, which impact drug efficacy and drug-induced toxicity in the use of warfarin maintenance dose, response to statin therapy, methotrexate toxicity, antipsychotic drugs and in hepatitis treatment [31]. Functional genomics and cell line model systems combined with clinical studies are useful for understanding pathways and novel mechanisms of drug response [31–33]. All this is being made possible by advances in genomic technology supported by the power of bioinformatics and systems approaches, integrating -OMICS sciences in pharmacogenomics [34–36]. Despite these advances in genomics technologies, which have enabled the transition from single marker analysis to genome-wide high-throughput methods at lower costs, the implementation of pharmacogenomics in clinical practice is still limited [37].

## Genetic variations influencing drug effects: strengthening the case for pharmacogenomic intervention

Earlier studies in pharmacogenetics focused on candidate genes known to catalyze well-delineated drug metabolic pathways. Some of the gene variants associated with drug metabolism in Asian and European populations are rare in African populations. However, some variants are common across world populations, although at different frequencies; for example, *GSTM1* deletion and *NAT2* slow variants. Studies carried out in African cohorts have identified some unique gene variants not reported in other world populations [38, 39].

Pharmacogenomics studies in African populations have focused on drugs used in the management of diseases that contribute to the high disease burden faced by sub-Saharan African populations, which include HIV, TB and malaria [40]. Genetic variations in genes coding for CYP enzymes have received the greatest attention because of their ubiquitous nature, broad substrate preferences and overlapping substrate specificities (Table 3) [5, 41, 42]. Cytochrome P450 enzymes are critical to successful therapy, and therefore are a major component of the very important pharmacogenetics genes and may hold the answer to some of the observed drug–drug interactions, since drugs indicated for different conditions could be under the metabolism of the same CYP enzyme.

**Table 3.** Commonly reported pharmacogenomics biomarkers

Drug (s)	Therapeutic area	Biomarker
Abacavir	Infectious Disease (anti-retroviral therapy)	*HLA-B*
Amitriptyline, Clozapine	Psychiatry	*CYP2D6*
Antidepressants	Depression	*CYP2D6*
Azathioprine	Rheumatology	*TMPT*
Artemether	Infectious disease (malaria)	*CYP2C8; CYP3A4; CYP3A5; CYP2B6*
Carbamazepine	Neurology	*HLA-B*
Carvedilol	Cardiology	*CYP2D6*
Celecoxib	Rheumatology	*CYP2C9*
Citalopram	Psychiatry	*CYP2C19; CYP2D6*
Chloroquine (plus Quinine, Quinidine)	Infectious disease (malaria)	*G6PD; CYP2D6*
Clopidogrel	Cardiology	*CYP2C19*
Clozapine	Psychiatry	*CYP2D6*
Codeine	Anesthesiology	*CYP2D6*
Efavirenz	Infectious disease (antiretroviral therapy)	*CYP2B6; CYP1A2; CYP2A6; UGT; CYP3A4*
Irinotecan	Oncology	*UGT1A1*
Isoniazid	Infectious Disease	*NAT2*
Metoprolol	Cardiology	*CYP2D6*
Rifampicin	Infectious disease (Anti-TB)	*CYP3A4*
Pravastatin, Simvastatin, Rosuvastatin	Endocrinology	*LDL-R; SLCO1B1; APOE; PCSK9*
Taclolimus	Organ transplantation	*CYP3A5*
Tamoxifen	Oncology	*ESR1; CYP2D6*
Thioguanide, Azathioprine	Oncology	*TMPT*
Tramadol	Analgesic	*CYP2D6*
Warfarin (Coumadin)	Cardiology or Hematology	*CYP2C9; VKORC1*

Globally, the most commonly studied genes associated with drug response include, but are not limited to, the cytochrome P450 genes (*CYP1A2, CYP2A6, CYP2B6, CYP2C8, CYP2C9, CYP2C19, CYP2D6* and *CYP3A4*), *VKORC1, SLC01B1, MDR-1* (*ABCB1*), *NAT-2* and *HLA*. Table 4 provides an analysis of a selected number of single nucleotide polymorphisms (SNPs) in genes that affect drug responses and from these few, it is clear that African populations are not fully characterized. There is a clear clustering of major world populations into Asian, Caucasian and African, extensively reviewed by Dandara *et al.* [5] and Alessandrini *et al*. [43], especially with regard to the genetic diversity of CYP alleles among African populations.

**Table 4.** Comparison of allele frequencies (%) of selected genetic variants as a measure of the importance of pharmacogenetics in different world populations

GENE	SNP/allele	Allele	Effect	CHB	JPY	CEU	Af.Am	MKK	TZA	ZWE	GHA	LWK	NGA	ZAF	ETH
*ABCB1*	*1236C>T (rs1128503)*		Null	62	59	38–54	6–14	14				14	12	9–13	
	*3435C>T (rs1045642)*		Null	58	46	46–57	7–17	57	16–22		11–17	84	21	12	15.5
	*4036A>G (rs3842)*		–		32	14	0		11				14	16–22	22
*CYP1A2*	*g-163C>A*	*1F	GoF	34	40	32	54	48	49	49	13	53	43	52	42–57
*CYP2A6*	*g.-48T>G*	*9	LoF	16	19	7	10				3–6	9	9	10	2.8
	*c.5065G>A*	*17	LoF		0	0.4	8–17				12	6	13–20	13–16	
*CYP2B6*	*c.516T*	*6	LoF	13–40	14–21	30	31–38	38	36–42	45–49	49	37	42	28–43	29–32
	*c.785G*	*4	GoF	10–28	19–27	14–37	35–46	35		51.4	48	37	22–45	36–41	30
	*c.983C*	*18	LoF	0	0	0.5	7.5–10	2	9.9	52.8	7.6	7	12	2.5–17	
*CYP2C8*	*c.805A>T*	*2	LoF	0	0	0	17		14–19		13–20		21		6.5
	*c.416G>A*	*3	LoF	1	0	10	1.1	2.5	0		0	2.5	0–2		3
	*c.792C>G*	*4	LoF	0	0	7	0.8		0.6		0		0		
*CYP2C9*	*c.430C>T*	*2	LoF	0	0	10	4–7		0	3	0	0	0	0	4
	*c.1075A>C*	*3	LoF	5	2	5.8	0.8		0	2	0	0	0	0	2
*CYP2C19*	*c.681G>A*	*2	LoF	37	35	15	25	11	18	13	3–9	11	10	21	4–11
	*c.636G>A*	*3	LoF	8	11	0	0	0	1	0		0	0	0	2
	*c.-806C>T; c.-340C>T*	*17	GoF	0–4	0–1.3	22–26	15–26						27		18
*CYP2D6*	*c.1846G>A*	*4		1	1	12–21	7	8	2	2	7–10	8	3	3	1–4
	Deletion	*5	LoF	6	3	2–7	6		4	4	6	–	–	5	1–3
	Duplication	*2XN		0.5	1	1–5			2	1	3	2	4	4	10–16
	*c.100C>T*	*10	LoF	51	43	0–2	4	5	4	6	3–12	5	7	12	3–9
	*c.1023C>T; c.2850C>T*	*17	LoF	0	0	0	15	18	18	35	15–28	18	22	24	3–9
	*c.1659G>A; 1661G>C; c.3183G>A*	*29	LoF	0	0	0	5	8	20	17	–	8	10	6	20
*CYP3A4*	*c.-392A>G (rs776746)*	*1B	GoF	0.5	0	3	68		74–78		65–78		79	66	52
	*c.522-191 C>T*	*22	LoF	0	0	5	1.1		0	0	1.2		0	0	
*CYP3A5*	*g.6986A>G*	*3	LoF	66	77	96	37	49	20	11.1	10–20	17	16	14	64
	*g.14690G>A*	*6	LoF	1.2	0.6	0	12		15–21		9–19		17	12	13
*GSTM1*	*Deletion*	*0	LoF	58	44	50	28		33	24	39	16	31	24	
*NAT2*	*c.341T>C*	*5	LoF	6	2	46	30	42	36	31		58	28–33	39	43
	*c.590G>A*	*6	LoF	31	23	29	22	27	27	21	27	24	27–33	22–34	35
	*c.857G>A*	*7	LoF	16	10	2.9	2	4	3	6	–	4	3	5	3.5
	*c.191G>A*	*14	LoF	0	0	0	9	9	13	14	10	9	8	11	
*SLC01B1*	*C.521T>C*	*5	LoF	13	11–16	10–15	2.3		0–2.9	0				0	21
*TPMT*	*c.248G>C*	*2	LoF	0	0	0.5	0.4				0	0			
*TPMT*	*c.460G>A; c.719A>G*	*3A	LoF	0	0	5.7	0.8				0	0			0
*TPMT*	*c.460G>A*	*3B/c	LoF	2.3	0.3	0.8	2.4				7.6	5.4		21	5.3
*UGT1A1*	*(TA)6>(TA)7*	*28	LoF	4–14	12	23–40					34				
*VKORC1*	*g.-1639A*		LoF	94	90	40	2.2							4	

CHB, Han Chinese from Beijing; CEU, Caucasian; ZWE, Zimbabwe (Shona); JPY, Japanese; Af.Am, African American: MKK, Masaai Kinyawa of Kenya; TZA, Tanzania; GHA, Ghana; LWK, Luhya of Webuwe Kenya; NGA, Nigeria (Yoruba, Ibo); ZAF, South Africa Bantu (Xhosa, Venda or Zulu); ETH, Ethiopia. LoF, loss-of-function; GoF, gain-of-function; CYP, cytochrome P450.

For a significant time, African governments have not actively funded research, and thus, local researchers have pursued the research goals of foreign funders. There are however promising efforts which have the potential to catalyze more extensive characterization of African genomes [5]. For example, the project, ‘RAFAgene’ [44], is investigating the contribution of genetic variations to pharmacokinetic variability and toxicity in patients undergoing treatment for multi-drug resistance tuberculosis in sub-Saharan Africa.

## HIV pharmacogenomics: the case of CYP2B6 genetic variation and the use of efavirenz and nevirapine

HIV infection and the need for its management remain huge challenges. To underscore the enormity of this challenge, there are at least 30 antiretroviral drugs approved for HIV, with no cure in sight. These antiretroviral drugs fall into five major types, as outlined below:

- *Entry Inhibitors*: act by interfering with viral binding to receptors on the outer surface of the cell.
- *Fusion Inhibitors*: mechanism of action involves interference with the ability of the virus to fuse with the cellular membrane, preventing HIV from entering a cell.
- *Reverse Transcriptase Inhibitors*: this class of drugs acts by preventing the reverse transcriptase enzyme from converting single-stranded HIV RNA into double-stranded HIV DNA and exists in two types, nucleoside/nucleotide reverse transcriptase inhibitors (NRTIs) and non-nucleoside reverse transcriptase inhibitors (NNRTIs).
- *Integrase Inhibitors*: their mechanism of action involves blocking the HIV integrase enzyme, which is used by the virus to integrate its genetic material into the DNA of the cell it has infected.
- *Protease Inhibitors (PIs)*: act by interfering with the function of the HIV enzyme called protease, which processes HIV polyproteins into mature, functional proteins.

The drugs fall into several classes depending on their mechanism of action and are used in a combination commonly referred to as highly active antiretroviral therapy (ART). Most of these drugs were developed based on studies conducted in European and Asian populations. Although HIV is such a huge burden across the African continent, of the 7183 clinical trials on HIV/ART, <14% (*n* = 983) involve participants of sub-Saharan African extraction (http://www.clinicaltrials.gov). Of the nearly six million individuals on ART world-wide, in 2012, a big proportion received combinations of two NRTIs and one NNRTI as initial first-line therapy and two NRTIs and one PI for patients on second line therapy [45]. The chronic nature of HIV treatment has led to new problems that impact on the quality of life of patients. Antiretroviral drug-associated side effects have become pronounced, mostly in patients on long-term treatment [24, 25, 46].

ADRs contribute to increased non-adherence to treatment by patients. Efavirenz (EFV) and nevirapine form the current backbone for first-line ART, despite many concerns related to their toxicity. A lot of research has focused on the pharmacogenetics of EFV. High interindividual variability in EFV plasma levels, after standard drug administration, has been reported. EFV levels >4 µg/mL have been linked to central nervous system (CNS)-associated side effects. EFV is primarily metabolized by *CYP2B6* to 8-hydroxy-EFV (8-OH-EFV), and other minor pathways involve *CYP1A2, CYP2D6, CYP3A4, CYP3A5* and *UGT2B7. CYP2B6* exhibits genetic polymorphisms, and nearly 40 alleles have been characterized to date (December 2015). Most of the CYP2B6 SNPs are associated with decreased CYP2B6 activity (http://www.cypalleles.ki.se). The most important variants in CYP2B6 include *CYP2B6 c.516G>T, c.785A>G* and *c.983T>C*, which have been widely characterized across world populations in terms of their effects on EFV plasma concentrations. EFV plasma concentrations <1 µg/mL are associated with virological failure, concentrations between 1 and 4 µg/mL represent the therapeutic range, while concentrations >4 µg/mL are associated with CNS-related side effects [47]. The *CYP2B6 c.516T* variant is associated with diminished CYP2B6 enzyme activity, and an increased incidence of EFV-associated neuropsychological toxicity [48]. Several studies have reported correlations between the CYP2B6 *c.516T/T* genotype and EFV plasma concentrations [25–27, 49, 50].

The frequency of the *CYP2B6 c.516T* variant is disproportionately distributed in world populations, occurring at frequencies of <30% among Europeans, <20% among Asians and >40% in some African populations. Thus, a large proportion of African patients are likely to present with CNS-associated side effects after taking EFV, compared with other world populations. The homozygous *CYP2B6 c.516T/T* genotype has also been linked to drug-induced liver injury in African patients, occurring at rates of 5% or greater in ART starters, and higher in those on co-treatment for tuberculosis. A standard dose of 600 mg/day is used and is mostly beneficial in European populations, with most patients achieving a therapeutic range, whereas in African populations, >50% of patients would present with EFV concentrations above 4 µg/mL [51]. To combat EFV toxicity in African patients, the findings of the ENCORE study [52], affirming the efficacy of a 400 mg/day EFV dose, should be adopted. Another important SNP to be monitored in EFV metabolism is *CYP2B6 c.983T>C*, which is also associated with decreased CYP2B6 enzyme activity [27, 53]. These loss-of-function mutations need to be characterized in African populations to reduce incidents of drug toxicity and ADRs.

## CYP2D6 and pharmacogenomics in Africans

Despite accounting for between 2% and 4% of all hepatic CYPs, CYP2D6 is one of the most widely studied CYP enzymes because it metabolizes nearly 25% of current therapeutic drugs. CYP2D6 metabolizes some of the most commonly used drugs that include antidepressants, antiemetics, beta-blockers and antipsychotics. There are more than 100 allelic variants reported for *CYP2D6* (http://www.cypalleles.ki.se) and a few are highlighted in Table 4. The majority of alleles result in large interindividual variability in CYP2D6 enzyme activity. CYP2D6 activity ranges considerably within a population and includes poor metabolizers (PMs), extensive metabolizers (EMs) [30], ultra-rapid metabolizers (UMs) and intermediate metabolizers (IMs).

The distribution of *CYP2D6* alleles in different populations shows both quantitative and qualitative differences. The spectrum of the alleles includes fully functional alleles, alleles coding for reduced function (differing at level of reduction) and non-functional alleles. Thus, a wide variety of drug responses (e.g. adverse drug effects) is likely to occur if standard doses are applied. Notably, some alleles associated with reduced enzyme activity act in a substrate dependent manner; for example, CYP2D6*17 affects some but not all substrates of CYP2D6 [54]. Alleles resulting in a complete absence of CYP2D6 activity make for easier predictions of phenotype and associations, and these include, *4, *5, *40, *44 and *56. In addition to alleles coding for an absence of *CYP2D6* activity, there are those coding for reduced activity and others associated with duplicated *CYP2D6*. Homozygosity or heterozygous constellations of *CYP2D6* alleles coding for abolished activity, lead to PMs, constellations of alleles coding for normal activity lead to EMs [30], combinations of abolished and reduced activity alleles lead to IMs, while homozygosity for duplications or constellations of duplications and normal activity variants lead to UMs [55].

*CYP2D6* genetic variation is important in African populations that are in health transition and with an increasing burden of non-communicable diseases. Most of the drugs metabolized by *CYP2D6* are indicated for treatment of cardiovascular related disorders and have narrow therapeutic indexes. In breast cancer therapy, tamoxifen has become an important drug in the treatment and prevention of recurrence in patients with estrogen receptor positive breast cancers; however, the generation of the potent metabolite, endoxifen, is dependent on *CYP2D6* metabolism. Thus, genetic variation in *CYP2D6* (e.g. PM status), has been associated with poor clinical outcomes in breast cancer patients treated with tamoxifen [56]. *CYP2D6* PM status varies between Europeans (<10%), Asians (up to 12%) and Africans (7%–19%). The different phenotypes associated with *CYP2D6* alleles are of clinical significance as they affect drug clearance and therefore resultant drug response. Across Africa, huge differences in the distribution of several *CYP2D6* variants can be observed. For example, CYP2D6*2xN ranges in frequency from as low as 1% to as high as 16%, and thus, marked differences in drug concentrations, as a result of the UM phenotype, are possible [57]. As mentioned earlier, CYP2D6*17 is an allele with a substrate-dependent reduced function, and its distribution among African populations can be as high as 35%, but is much lower among Ethiopians (<10%) and generally absent among Asian populations (0%). This highlights the fact that as much as there are differences in the distribution of variant alleles between major world populations (Caucasian, Asian and African), there are also significant differences within African populations. Thus, treatment with drugs that are substrates of CYP2D6 is likely to result in ADRs or altered drug responses, in some African populations because of these differences in the distribution of allelic variants. Many studies have shown *CYP2D6* genotype and phenotype association with respect to particular drugs [16, 58]; however, genotype testing for CYP2D6 has not yet been accepted in clinical practice.

## CYP3A4 and CYP3A5 pharmacogenetics in African populations

The CYPs 3A4 and 3A5 are important enzymes because of the wide spectrum of drugs they metabolize. Population variability in CYP3A4 and CYP3A5 activity is extremely high, sometimes >100-fold. The variation in activity or expression levels is attributed to both environmental and genetic factors. The most commonly reported SNPs in *CYP3A4* include CYP3A4*1B (*CYP3A4 g.-392G>A)* and CYP3A4*22 (15389 C>T; rs35599367). CYP3A4*1B affects the expression levels of *CYP3A4* and its allele frequency distribution varies between different populations, occurring at 4% in Europeans and up to 82% among Africans. CYP3A4*22 has been associated with decreased expression and activity (up to fivefold less), occurring in nearly 2% of Europeans, but it is rare among African populations [59].

CYP3A5 demonstrates highly variable protein expression, which is mostly attributed to three SNPs; *CYP3A5 c.6986A>G, c.14690G>A* and *27131_27132insT*, coding for CYP3A5*3, CYP3A5*6 and CYP3A5*7 alleles, respectively. CYP3A5*3/*3 homozygosity is associated with the absence of CYP3A5 activity and this genotype frequency differs among world populations. Among Europeans, the frequency ranges from 80% to 90%, and it varies widely among African populations (Ghana 4%, Senegal 9%, Malawi 5%, Nigeria 2% and Ethiopia 53%)[42]. The other two alleles, CYP3A5*6 and CYP3A5*7, are not as present as frequently, and appear to be of recent African ancestry. These marked differences across populations underscore the need for population-based pharmacogenetics, especially within the African continent, with its diverse genetic variability.

## Pharmacogenomics in cardiovascular disease (CVD)

Africa is experiencing a steep increase in the reported levels of CVD because of a combination of both improved health awareness, leading to increased disease detection and changing health dynamics. According to the World Health Organization, CVDs [60] are the leading cause of death globally, contributing to approximately 17 million deaths annually [60]. The sub-Saharan CVD epidemic is driven partly by hypertension, which remains the major risk factor for stroke, with at least 50% of adults >25 years estimated to be hypertensive [61]. Changing lifestyles in sub-Saharan Africa have increased the exposure to risk factors for CVD, including tobacco smoking, high-fat diets and physical inactivity. This increased disease burden has stretched the treatment requirements and needs of African patients. CYP2D6 and CYP2C19 are the major enzymes involved in the oxidative metabolism of most anti-hypertensives, antidepressants and *β*-blockers. Genome-wide association studies have unlocked the genetic basis of some of the adverse drug events experienced by people taking anti-hypertensives, although only a few such studies have included patients with sub-Saharan African ancestry [7].

Clopidogrel and warfarin represent two of the most successful stories of how pharmacogenetics has influenced treatment of CVD in clinical practice. However, this success is not shared among African populations as the genetic variants affecting drug dosing in other populations are rare in African populations [62]. Clopidogrel is an antiplatelet agent administered for the treatment of patients undergoing percutaneous coronary interventions [62, 63]. Clopidogrel is administered as an inactive pro-drug that requires oxidation by the CYP system to an active metabolite and its use is complicated by interactions with many other therapeutic drugs [63]. CYP2C19 is one of the principal enzymes involved in the bioactivation of the clopidogrel antiplatelet prodrug (Table 3). CYP2C19 is highly polymorphic, with the most common loss-of-function variant, CYP2C19*2, having a frequency of 25%–30% (European and African-Americans) and up to 70% in Asians. Among Africans, the frequency of the loss-of-function homozygous genotypes ranges from 14% to 20% [13]. The differences in the distribution of the loss of function variants result in differences in the way patients from different populations respond to medication metabolized by CYP2C19. Thus, these observations led the FDA to issue a warning on the label for clopidogrel, although there are conflicting reports on the clinical utility of CYP2C19 genotyping-based prescriptions [64].

Warfarin is the most commonly used anticoagulant and from the first few days up to 6 months of starting treatment, it is associated with hospitalization in 20% of patients. Warfarin exerts its anticoagulant effects through inhibiting the protein vitamin K epoxide reductase complex, subunit 1 (VKORC1), which is an important determinant of warfarin response. A VKORC1 promoter polymorphism (*VKORC1 g.–1639G>A*), especially in the homozygous form, the *VKORC1 g.–1639A/A* genotype, is significantly associated with warfarin-sensitivity [65]. Warfarin is principally metabolized by CYP2C9, with more than 60 alleles reported. The main variants include CYP2C9*2 and *3, of which homozygosity for either CYP2C9*2 and *3 or heterozygosity for non-functional alleles is associated with <10% CYP2C9 enzyme activity. However, these two variants are rare in African-Americans and not much known about their frequencies in various African populations [66, 67]. Reports in European populations show that prior pharmacogenomics testing for CYP2C9 variants coupled with dosage adjustments result in reduced hospitalization [68]. Thus, the combined contribution of genetic variation in both CYP2C9 and VKORC1 accounts for nearly 40% of the interindividual variability in warfarin dose requirements among Caucasians [69]. As explained in Dandara *et al*. [5], there is a need for further characterization of African populations with respect to warfarin response [70].

**Fig. 1.**
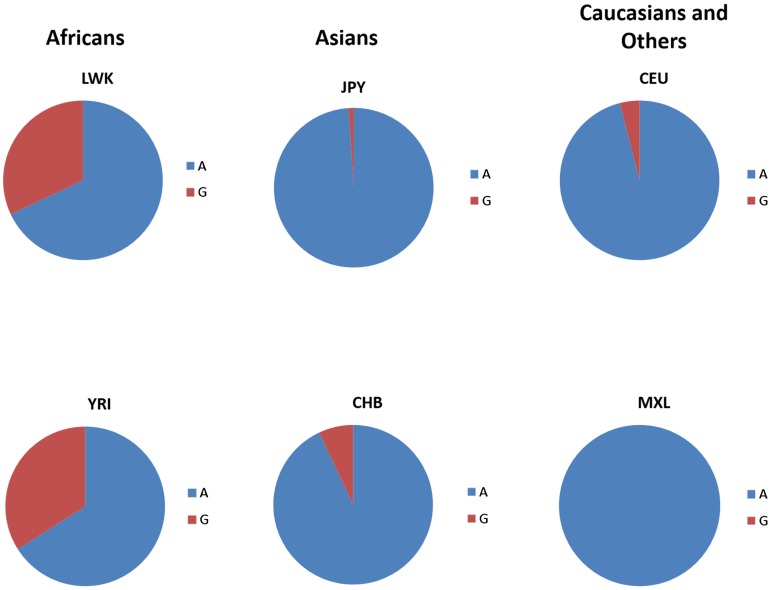
Distribution of *PCSK9* rs505151 allele frequencies in selected world populations. The *PCSK9* rs505151(G) variant, depicted in red, is a gain-of-function mutation that is also associated with increased risk for coronary artery disease. The rs505151(A) variant is depicted in blue. Data from studies in African, Asian and other world populations were obtained from 1000 Genomes Project (http://www.1000genomes.org/) and HapMap (http://hapmap.ncbi.nlm.nih.gov/). Reviewed populations included: LWK, Luhya in Webuye, Kenya; YRI, Yoruba from Ibdan, Nigeria; CHB, Han Chinese in Beijing, China; JPY, Japanese; CEU, Utah residents with Northern and Western European ancestry; MXL, Mexican Ancestry in Los Angeles, California.

With an increasing incidence of non-communicable diseases, statins, a class of lipid-lowering drugs, have become a commonly used treatment. Statins present with other pleiotropic effects, such as anti-inflammatory and anti-oxidative effects [71]. Although statins have outperformed other lipid lowering drugs since their release [72], they present with side effects in up to 10% of patients [73]. A common non-synonymous polymorphism in *PCSK9*, E670G (*rs505151A>G*), has been associated with reduced levels of low-density lipoprotein receptor (LDLR), which increases blood cholesterol levels and has been associated with a poor LDL-C response to statin treatment [71]. PCSK9 binds to LDLR at the cell surface, disrupting its normal recycling and therefore gain-of-function mutations have the effect of maintaining high levels of serum LDL-C. The *PCSK9 rs505151G* variant is a gain-of-function mutation and is associated with increased risks for coronary artery disease and ischemic stroke [74]. Data from the 1000 genomes project show that the *PCSK9 rs505151G* variant is more prevalent in populations with African ancestry such as African-Caribbeans in Barbados (25%), the Mandika of Gambia (26%), and the Yoruba of Nigeria (34%), and is rare among the Japanese (1%), and absent among populations with Mexican ancestry in Los Angeles, California (0%) (Fig. 1). Statins have generally received a favorable safety profile in clinical trials. However, they may not be as safe in African populations, if the *PCSK9 rs505151* SNP distribution data are considered. In addition to *PCSK9*, kinesin-like family member 6 (*KIF6*) also acts as a prognostic marker of statin efficacy [73], through its role in intracellular transport of mRNAs, protein complexes and organelles. Carriers of the rs20455C (*2155T>C*) variant display an increased risk for coronary events, as well as greater benefit from statin therapy. The rs20455 SNP, a putative risk factor for coronary heart disease, also shows tremendous variation among world populations and therefore it is likely to affect responses to statin medication [75]. Through these examples, a strong case for the inclusion of African patients or participants in clinical trials can be made.

## Malaria and sickle cell disease (SCD): role for pharmacogenomics interventions

Malaria, caused by *plasmodium falciparum*, is one of the leading causes of death in Africa (http://www.who.int/malaria). The efficacy of anti-malarial drugs is affected by host variability in drug metabolism. Genetic polymorphisms in drug metabolizing enzymes may be associated with treatment failure [76]. For the treatment of uncomplicated malaria, artemisinin-based combinations are used, and these are mostly metabolized by CYP2C8, CYP2B6, CYP3A4 and CYP3A5. Variants such as CYP2C8*2, CYP2C8*3, CYP2B6*6, CYP3A4*1B and CYP3A5*3 may affect an individual's response to treatment. CYP2C8 variants have been poorly characterized in African populations. However, in the few studies that exist, their frequencies vary between 0% and 4% [77–79]. CYP2B6*6 and CYP3A4*1B variants occur with significant variation in African populations, and range from 22% to 51% and 50% to 80%, respectively [80]. This variation is likely to manifest as a differential drug response phenotype.

Malaria and SCD are major health problems among African populations. Approximately 300 000 children are born with SCD every year world-wide, 75% of whom are in sub-Saharan Africa [81]. The geographical distribution of sickle cell trait mirrors that of malaria [82–84]. In Africa, the highest prevalence of sickle cell trait occurs in countries such as Cameroon, Republic of Congo, Gabon, Ghana, Nigeria and Uganda, ranging from 10% to 45% of the population [85–87]. Complications of SCD include attacks of severe pain, pulmonary hypertension and increased risk of early death. The pharmacological management of SCD includes use of preventive antibiotics, analgesics to relieve pain, blood transfusion and the drug hydroxyurea [82]. Hydroxyurea (HU) is the only drug currently approved by the FDA for the treatment of SCD. It is effective in increasing total and fetal hemoglobin (HbF), with a consequent reduction in the incidence of acute pain episodes, acute chest syndrome, and hospitalization [88]. Despite its proven efficacy, HU-induced increases in HbF show wide interindividual variability [6, 89].

It is imperative that more studies, especially pharmacogenomics studies, are carried out to evaluate the genomic markers of optimal responses for HU, especially among African populations [90]. To date there are no studies that have examined the pharmacogenomics of HU in African populations [91–93]. A whole exon sequencing genotype–phenotype association study identified a variant located in the *SALL2* gene, which was associated with HU response. The *P840R* variant in the *SALL2* gene (rs61743453) was associated with a higher change in HbF in response to HU in both the discovery and validation cohorts.

## Summary

This paper has reviewed the literature on pharmacogenomics data in African populations and outlines the following imperative points to make a case for pharmacogenomics intervention in African populations:

- The people of Africa are not genetically homogenous. Various ethnic groups inhabit the continent, with a wide genetic diversity.
- Africa faces a huge disease burden with a multiplicity of co-morbidities that require many therapeutic drugs for treatment and this creates a challenge in terms of drug–drug interactions.
- African populations possess some unique genetic markers that affect the way they respond to therapeutic drugs, which if identified, may contribute to a precision medicine approach to treatment and consequently a reduction in drug-associated side effects.
- It should also be recognized that some existing recommendations for the use of certain drugs might not be useful to selected African populations because of the absence of known variants or the presence of ethnic-specific variants.
- Identification/discovery of genetic markers of drug response in African populations could be of global value because the genomes of African populations represent the human ancestral origin of the world population.

## Conclusion

Pharmacogenomics research is critical for the improvement of health in Africa. Understanding how diseases interact, as well as how treatments for different diseases are likely to affect each other, together with the underlying genetic variation of patients, will play a major role in combating the disease burden in African patients. Although we have concentrated on variations in genes encoding proteins involved in PK and PD, it is important to understand that variations in mitochondrial DNA also play an important role in drug response. The biggest challenge in disease management in African patients is co-infections (e.g. malaria, TB, HIV and schistosomiasis), which require multiple concurrent medications. Such co-morbidities differ with geographical region in Africa. As a population in healthcare transition, supplementary treatments using herbal and over-the-counter products are prevalent among African patients. Therefore, unlike other world populations, where specific drugs are used for the management of diseases and where infectious diseases have become a rarity, Africa and its people are likely to benefit the most from pharmacogenomics studies. There is a need to characterize major ethnic groups with respect to the genetic determinants of drug response. Unraveling the pharmacogenomics of African populations is likely to not only being helpful for African patients, but also to most other world populations, because of the ‘out-of-Africa’ basis for human diversity. Precision medicine, which incorporates knowledge of genetic variation and environmental interactions, should usher in a new approach in treating patients on the African continent. Genomics will play a significant role in the optimization of the benefit-risk ratio of therapeutic drugs, thereby maximizing efficacy and safety, and minimizing toxicity. Africa could benefit more from pharmacogenomics if African populations are routinely included in drug-validating clinical trials that form the basis of drug development.

Recommendations to promote pharmacogenomics research in Africa

- More resources should be made available to extensively characterize the genetic diversity of major ethnic groups in each African country.
- Pharmacogenomics research should be coordinated so that there is harmonization in genetic characterization and phenotyping methodology; this could be achieved through the ‘African Pharmacogenomics Consortium’ formed during the first Pharmacogenetics and Precision Medicine Conference in Africa, in Cape Town (7–9 April 2016) and consolidated during the 9th Congress of the African Society of Human Genetics (Dakar, Senegal, May 2016).
- Parallel research investigating commonly used herbal medicinal plants and their characterization for interaction with conventional medicines should be undertaken.
- Strengthening the ethical, legal and social issues associated with genomic research should be prioritized and in particular issues regarding consent, incidental genetic findings and data sharing.
- Training of postgraduate students in genomics research, including genomic data handling and analysis should be an important component of future pharmacogenomics endeavors.
- Educating physicians and the public on the utility of pharmacogenomics and its possible impact in healthcare will lead to acceptance in clinical practice.
